# Profiling of Volatile Compounds and Associated Gene Expression in Two *Anthurium* Cultivars and Their F1 Hybrid Progenies

**DOI:** 10.3390/molecules26102902

**Published:** 2021-05-13

**Authors:** Qian Wei, Qing Xia, Yue Wang, Wen Chen, Cuiling Liu, Ruizhen Zeng, Li Xie, Maosheng Yi, Herong Guo

**Affiliations:** 1 Guangdong Key Laboratory for Innovative Development and Utilization of Forest Plant Germplasm, College of Forestry and Landscape Architecture, South China Agricultural University, Guangzhou 510642, China; weiqian@scau.edu.cn (Q.W.); wy-7477@stu.scau.edu.cn (Y.W.); huasheng@szhsco.cn (W.C.); cuilingliu@stu.scau.edu.cn (C.L.); zengrz@scau.edu.cn (R.Z.); xieli@scau.edu.cn (L.X.); 2 Guangzhou Flower Research Center, Guangzhou 510360, China; yuqingxia5565@126.com

**Keywords:** *Anthurium andraeanum*, hybrid progenies, floral scent, VOCs, gene expression

## Abstract

*Anthurium* is an important ornamental crop in the world market and its floral scent can enhance its ornamental value. To date, studies of the components and formation mechanism of the floral scent of *Anthurium* are relatively few. In this study, the scent profiles of two *Anthurium* varieties were measured by gas chromatograph-mass spectrometer (GC-MS). There were 32 volatile organic compounds (VOCs) identified in *Anthurium* ‘*Mystral’*, and the most abundant compound was eucalyptol (57.5%). Extremely small amounts of VOCs were detected in *Anthurium* ‘Alabama’. Compared with *A.* ‘Alabama’, most genes related to floral scent synthesis exhibited a higher expression in *A.* *‘Mystral’*, including *AaDXS, AaDXR*, *AaMDS*, *AaHDS, AaTPS, AaDAHPS,* *AaADT2*, *AaPAL1*, and *AaPAL2.* In order to produce new varieties of *Anthurium* with fragrance, 454 progenies of two crossbred combinations of *A.* ‘*Mystral*’ and *A.* ‘Alabama’ were obtained. Four F1 generation plants with different floral scent intensities were selected for further study. The major components of floral scent in the progenies were similar to that of the parental *A.* *‘Mystral’* plant. The expression patterns of genes related to floral scent synthesis were consistent with the relative contents of different types of VOCs. This study revealed the profiles of volatile compounds and associated gene expression in two *Anthurium* cultivars and their F1 hybrids, which provided a basis for the floral scent inheritance of *Anthurium andraeanum*.

## 1. Introduction

The floral scent is an important trait of ornamental plants and plays a crucial role in attracting pollinators [1] and pathogen resistance [2]. In addition, flower scent can attract customers and improve the commercial potential of an ornamental plant [3]. The floral scent is composed of a series of low-molecular-weight volatile organic compounds (VOCs). To date, VOC profiles have been analyzed in many species, including *Dianthus caryophyllus* L. [4], *Rosa ×* *hybrida* [5], *Osmanthus fragrans* Lour. [6], *Freesia* [7], *Lycoris* [8], *Gelsemium sempervirens* [9], *Lilium* [10], *Chimonanthus praecox* [11], *Polianthes tuberosa* L. [12], *Prunus mume* [3], and *Freesia hybrida* [13], amongst others. Although the VOCs emitted by flowers vary greatly among different species, they can be divided into three major groups according to their biosynthesis origins: terpenoids, phenylpropanoids/benzenoids, and fatty acid derivatives [14].

**Figure 1:**
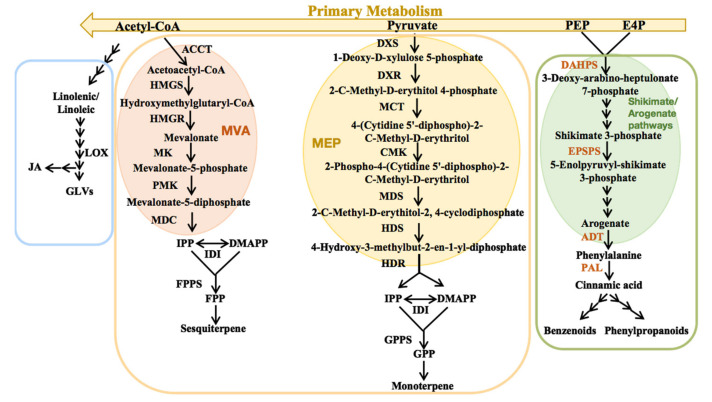
Overview of the main volatile organic compounds’ biosynthetic pathways: terpenoids (pink), phenylpropanoids/benzenoids (blue), and fatty acid derivatives (yellow).In this study, the components of floral scents and the expression levels of floral scent biosynthesis-related genes were identified in *A. ‘Mystral’* (with strong fragrance) and *A.* ‘Alabama’ (with no fragrance). In addition, the floral scent biosynthesis characteristics were further explored in the F1 hybrids of *A. ‘Mystral’* and *A.* ‘Alabama’, including the presence or absence of floral scent, the types and contents of VOCs, and the expression patterns of VOC synthesis-related genes. In this study, the hybrid progenies of *Anthurium andraeanum* with aroma and *Anthurium andraeanum* without aroma were established and the inheritance of aroma was preliminarily explored, providing a theoretical basis for the inheritance of floral scent in *A. andraeanum* and laying a foundation for the creation of new *A. andraeanum* varieties with fragrance.

Terpenoids are the largest class of plant VOCs and their metabolic pathways have been well characterized in the plant kingdom (Figure 1). Terpenoids are synthesized via the cytosolic mevalonate acid (MVA) pathway and the plastidial methylerythritol phosphate (MEP) pathway [15]. The MVA pathway begins with the condensation of three molecules of acetyl-CoA, whereas the MEP pathway starts with the condensation of D-glyceraldehyde 3-phosphate and pyruvate. Both pathways generate isopentenyl diphosphate (IPP) and its homologous isomer dimethylallyl pyrophosphate (DMAPP) through a series of enzymatic reactions [16]. The sequential head-to-tail condensation of IPP and DMAPP leads to the formation of FPP in the cytosol, as well as pyrophosphate (GPP) and geranylgeranyl diphosphate (GGPP) in plastids [17]. Subsequently, different terpene synthases (TPSs) convert GPP, GGPP, and FPP into structurally diverse monoterpenes, diterpenes, and sesquiterpenes, respectively [18]. The monoterpenes are mainly synthesized by the MEP pathway, in which nine key enzymes catalyze a series of successive reactions, including 1-deoxy-*D*-xylulose 5-phosphate synthase (DXS), 1-deoxy-*D*-xylulose 5-phosphate reductoisomerase (DXR), 2-C-methyl-*D*-erythritol 4-phosphate cytidylyl transferase (MCT), 4-(cytidine 5′-diphospho)-2-C-methyl-*D*-erythritol kinase (CMK), 2-C-methyl-Derythritol-2,4-cyclodiphosphate synthase (MDS), 4-hydroxy-3-methylbut-2-en-1-yl diphosphate synthase (HDS), 4-hydroxy-3-methylbut-2-en-1-yl diphosphate reductase (HDR), geranyl pyrophosphate synthase (GPPS), and TPS [19]. Phenylpropanoids and benzenoids, the second largest class of plant VOCs, are divided into three subclasses depending on their carbon skeleton: phenylpropanoids (with a C6–C3 backbone), benzenoids (with a C6–C1 backbone), and phenylpropanoid-related compounds (with a C6–C2 backbone) [14]. All three subclasses are derived from the aromatic amino acid (AAAS) phenylalanine (Phe). Aromatic amino acid aminotransferase (AAAT) and phenylacetaldehyde synthase (PAAS) are key enzymes in phenylpropanoid-related compound biosynthesis, and phenylalanine ammonia-lyase (PAL) is the key enzyme in benzenoid and phenylpropanoid biosynthesis, which deaminates Phe to *trans*-cinnamic acid (CA) and competes with PAAS for Phe utilization. Phe is derived from chorismate, the final product of the shikimate pathway [20]. Many studies have demonstrated that the level of Phe is greatly regulated by the activity of key enzymes in the shikimate and arogenate pathways (Figure 1), such as 3-deoxy-di-arabino-heptulosonate 7-phosphate synthase (DAHPS), 5-enolpyruvylshikimate-3-phosphate synthase (EPSPS), chorismate mutase (CM), prephenate dehydratase (PDT), arogenate dehydratase (ADT), and S-adenosylmethionine synthetase (SAMS) [21,22]. Fatty acid derivatives, the third class of plant VOCs, are derived from unsaturated C18 fatty acids, namely linolenic or linoleic acids [23]. Lipoxygenase (LOX) directly leads to the formation of 9- and 13-hydroperoxy intermediates via two branches: the allene oxide synthase (AOS) branch, which gives rise to jasmonic acid (JA), and the hydroperoxide lyase branch, which leads to the formation of volatile alcohols and their esters [24].

Many transcription factor (TF) families are involved in regulating the biosynthesis of VOCs. The *TPS* gene family, responsible for the formation of terpenes, has been characterized in many plants—however, the regulatory network that controls *TPS* expression is still unclear. In *Arabidopsis thaliana* (Arabidopsis), myeloblastosis protein 21 (MYB21) and myelocytomatosis protein 2 (MYC2) regulate the expression of *TPS11* and *TPS21* via the gibberellic and JA pathways [25,26]. In Arabidopsis, auxin response factors 6 and 8 (ARF6 and ARF8) can bind to the promoters of *TPS11* and *TPS21* to regulate the synthesis of sesquiterpenes [25]. In addition, MYB TFs can interact with other base helix–loop–helix (bHLH) TFs to form an MYB–bHLH complex to participate in the regulation of sesquiterpene biosynthesis [27,28]. WRKY TFs, NaWRKY3, and NaWRKY6 participate in the regulation of the defensive terpene emission in *Nicotiana attenuata* [29]. In *Petunia × hybrida*, R2R3-type MYB TFs, ODORANT1 (ODO1), EMISSION OF BENZENOIDS I (EBOI), and EMISSION OF BENZENOIDS II (EBOII) control the biosynthesis of phenylpropanoids/benzenoids by regulation of the shikimate pathway [30,31]. Suppression of *PhODO1* and *PhEBOII* expression leads to a reduced amount of floral volatiles, through decreasing transcript level of many phenylpropanoid/benzenoid genes such as *PhDAHPS, PhEPSPS*, *PhPAL*, *PhCM,* and *PhSAMS* [32]. In contrast, the MYB4 TF is a repressor of cinnamate-4-hydroxylase from the phenylpropanoid pathway in petunias [33]. For the biosynthetic pathway of the volatiles derived from fatty acids, studies on their transcriptional regulation have primarily focused on JA [34,35]. Many TFs, including MYC, MYB, GAI, RGA, EIN3, EIL, ERF, and RGL1, have been demonstrated to be involved in the regulation of JA biosynthesis [23,36].

*Anthurium andraeanum* is an important tropical and subtropical ornamental crop in the world market. The popularity of *A. andraeanum* is largely due to the exotic shapes and colors of the spathe, and the remarkable longevity of the plant’s flowering period. The floral scent varies greatly among Anthurium cultivars. A survey of floral scent in 147 Anthurium species and hybrids showed that most plants emitted scents ranging from pleasant to unpleasant and from very weak to very strong [37]. The majority of Anthurium species that emit fragrance and volatile compounds do so at the pistillate stage of flower development, with the primary emitted compounds consisting of 1,8-cineole, α,β -pinene, sabinene, myrcene, and limonene, as well as some benzenoids [37]. To date, studies characterizing the components and formation mechanism of Anthurium floral scent are scarce.

## 2. Results

### 2.1. Floral Scent Compounds in the Spadix of A. ‘Mystral’ and A. ‘*Alabama*’

**Figure 2:**
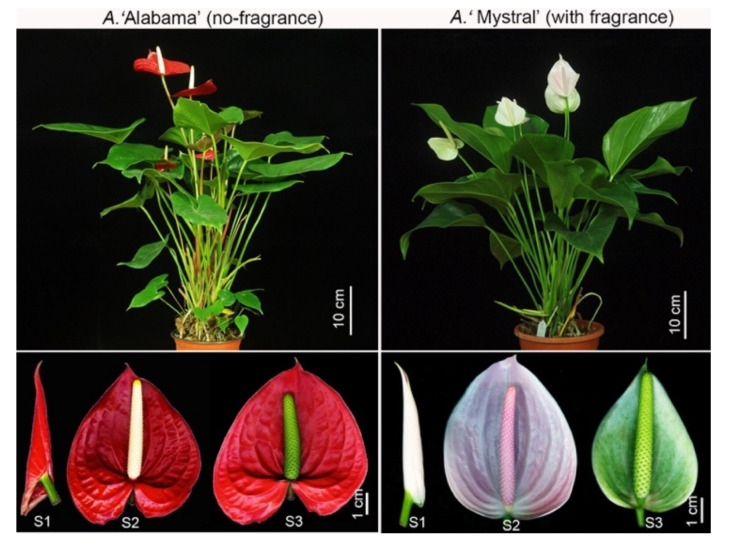
Phenotype of *A.* ‘Alabama’ and *A.* ‘*Mystral*’. S1, spathe folding stage; S2, pistillate emerge stage; S3, spadix fully extended stage.

*A.‘Mystral’* (with strong fragrance) and *A.* ‘Alabama’ (with no fragrance) were the best-selling varieties of *Anthurium*, and were selected for floral scent study (Figure 2). To identify the components of the floral scent of *Anthurium*, total VOCs from the spadix in fully expended stage (S2) were analyzed (Figure 2) by gas chromatograph-mass spectrometer (GC-MS). In *A.* *‘Mystral’*, 32 VOCs were identified (Table 1), including terpenes (70%) and phenylpropanoid/benzenoids (28.5%). The total amount of VOCs was 85.567 µg·h^−1^·g^−1^, with eucalyptol (49.2 µg·h^−1^·g^−1^) and acetic acid, phenylmethyl ester (19.835 µg·h^−1^·g^−1^) being the predominant components, accounting for 57.5% and 23.2% of the total VOCs, respectively. Compared with *A.* *‘Mystral’*, only 1.376 µg·h^−1^·g^−1^ VOCs were detected in *A.* ‘Alabama’, and no terpenes or phenylpropanoid/benzenoids were identified. These results were consistent with the sensory judgment, and indicated eucalyptol was the major component of the floral scent of *A. ‘Mystral’*.

### 2.2. Analysis of the Key Genes Involved in Volatile Organic Compound (VOC) Biosynthetic Pathways

**Figure 3:**
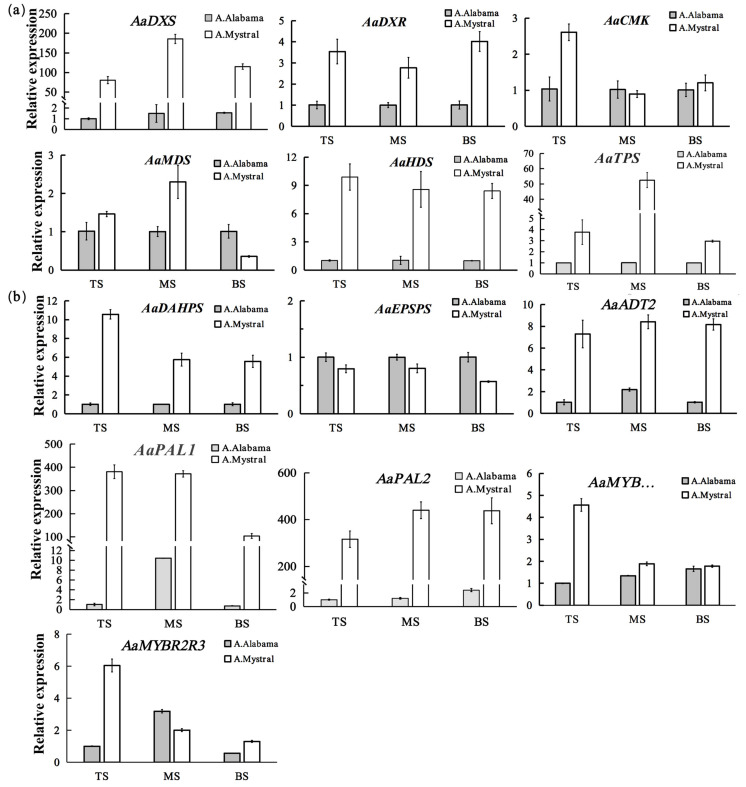
The relative expression levels of VOC biosynthesis-related genes in the inflorescences of *A.* *’Mystral’* and *A.* ‘Alabama’. (**a**) The relative expression levels of monoterpene biosynthesis-related genes; (**b**) The relative expression levels of key genes in the phenylpropane biosynthesis or shikimate pathways. TS, the top part of the inflorescences; MS, the middle part of the inflorescences; BS, the base part of the inflorescences.

In most flowering plants, VOCs are divided into several classes, including terpenoids, benzenes/phenylpropanes, and fatty acid derivatives. In the 32 VOCs identified in *A. ‘Mystral’*, 13 of these were monoterpenes while six were benzenes/phenylpropanes. To further characterize the regulation of VOCs at the molecular level, the transcript levels of key genes involved in monoterpene and phenylpropane biosynthesis were assessed (Figure 3). In total, 10 related genes were retrieved from NCBI and our transcriptome database (unpublished), including six key genes in the MEP pathway (*AaDXS, AaDXR*, *AaCMK*, *AaMDS*, *AaHDS,* and *AaTPS*) and five key genes in the phenylpropane biosynthesis or shikimate pathways (*AaDAHPS, AaEPSPS*, *AaADT2*, *AaPAL1,* and *AaPAL2*). In *A.* ‘Alabama’, most VOC biosynthesis-related genes showed similar expression patterns in all three parts of the inflorescence, except for *AaPAL1*. The expression level of *AaPAL1* in the middle part of the spadix (MS) was ten times higher than in the top part of the spadix (TS) and the base part of the spadix (BS). Unlike in *A.* ‘Alabama’, most VOC biosynthesis-related genes showed higher expression in the TS of *A.* *‘Mystral’*. Compared with the TS of *A.* ‘Alabama’, the expression level of *AaDXS*—the first enzyme in the MEP pathway—increased at least 80-fold in all three parts of the *A.* *‘Mystral’* spadix. *AaTPS*, the key enzyme responsible for production of the major monoterpenes, showed a 52-fold increase in the BS of *A.* *‘Mystral’*. The expression levels of *AaDXR* and *AaHDS* were much higher in all three parts of the *A.* *‘Mystral’* spadix. Compared with the TS of *A.* ‘Alabama’, *AaCMK* and *AaMDS* exhibited higher expression both in the TS and in the MS of *A.* *‘Mystral’*. *AaDAHPS* and *AaEPSPS* were the key enzymes in the shikimate pathway assessed in this study, and the expression of *AaDAHPS* was higher in *A.* *‘Mystral’* spadix than in *A.* ‘Alabama’ spadix, while the expression level of *AaEPSPS* was not significantly different between the two cultivars. Both *AaPAL* and *AaADT*, the key enzymes in the phenylpropane biosynthesis pathway, showed higher expression in *A.* *‘Mystral’* than in *A.* ‘Alabama’ spadix. These results indicated that the expression levels of VOC biosynthetic pathway-related genes were significantly different between *A.* *‘Mystral’* and *A.* ‘Alabama’, and *AaDXS*, *AaTPS,* and *AaPAL* might be the key functional genes in the biosynthesis of VOCs.

### 2.3. Segregation of Floral Scent Traits in Hybrid Progenies of A. ‘Mystral’ × A. ‘*Alabama*’

In order to determine whether floral scent traits can be inherited or not, the floral scents of F1 hybrids resulting from the genetic crossing of *A*. *’Mystral’* and *A.* ‘Alabama’ were studied. The olfactory tests were performed by treated individuals [13]. The results showed that the proportion of fragrant plants of F1 generation from the cross (*A.* *’Mystral’* ♀ × *A.* ‘Alabama’ ♂) or the reciprocal cross (*A.* *’Mystral’* ♂ × *A.* ‘Alabama’ ♀) were 74.38% and 59.34%, respectively (Table 2). The fragrant plants were classified according to floral scent intensity. There were 60 plants with strong fragrance and 210 plants with weak fragrance in F1 populations from cross combination, and the ratio of fragrant plants to fragrance-free plants was 3:1. In F1 populations from reciprocal cross combination, six plants were identified as strong fragrant plants, 48 plants were weak fragrant plants, and the ratio was 3:2. These data indicate that the floral scent is a complex trait and is likely to have many genes of influence.

### 2.4. The Compounds of Floral Scent in Hybrid Progenies of A. ‘Mystral’ × A. ‘*Alabama*’

Several individual plants with different floral scent intensities in the hybrid progenies were selected to test the VOCs (Table 3). Progeny plants 08-377-09 and 08-382-20 were strong fragrant plants, while progeny plants 08-377-03 and 08-382-48 were weak fragrant plants. In total, 27 VOCs were identified in plant 08-377-09, and the relative total amount of VOCs was 109.811 µg·h^−1^·g^−1^, including terpenes (10.8%), benzenoids (84.5%), and fatty acid derivatives (4.2%). Unlike the fragrant parental *A.* *‘Mystral’*, acetic acid, phenylmethyl ester was the most abundant component, and the content of eucalyptol was not as high as that of *A.* ‘*Mystral*’. In plant 08-382-20, the total amount of VOCs was 20 with a relative total amount of 113.137 µg·h^−1^·g^−1^, including 15 terpenes that accounted for 90% of the total VOCs. Eucalyptol (33%) was the major compound in plant 08-382-20, followed by 3,7-dimethyl-1,6-Octadien-3-ol (24%), *trans*-Limonene oxide (10%), *trans*-Carvone oxide (9%), and *trans*-2-methyl-5-(1-methylethenyl)-Cyclohexanone (9%). For the plants 08-377-03 and 08-382-48, the total amounts of VOCs were relatively less, and the most abundant components were acetic acid, phenylmethyl ester, and eucalyptol, respectively. These results indicated that the floral scent traits of *Anthurium* can be inherited, and the major floral scent components were similar to the fragrant parental plants.

### 2.5. Analysis of Volatile Organic Compound Biosynthetic Pathway-Related Genes in Hybrid Progenies

**Figure 4:**
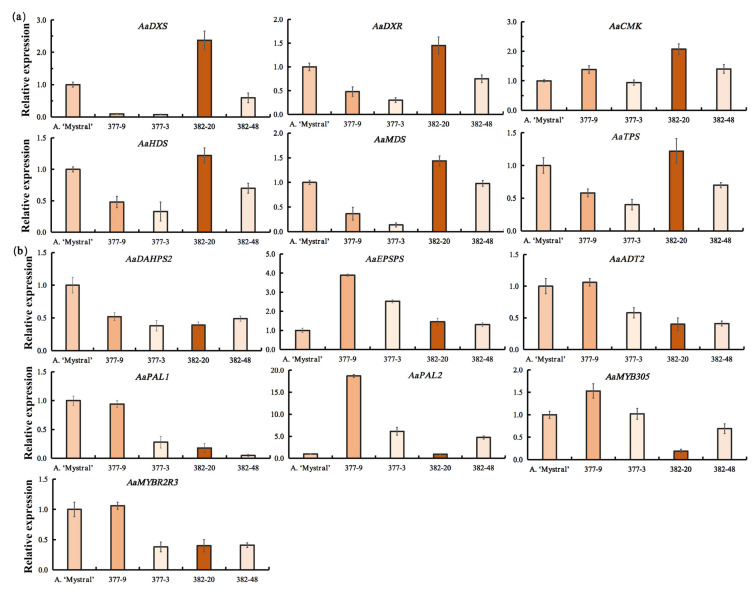
The relative expression levels of VOC biosynthesis-related genes in the top part of the inflorescences of progenies and *A. ‘Mystral’*. (**a**) The relative expression levels of monoterpene biosynthesis-related genes; (**b**) The relative expression levels of key genes in phenylpropane biosynthesis or shikimate pathways. The relative bar color intensities represent the total amount of VOCs.

To explore the relationship between floral scent compounds and VOC biosynthesis-related genes in these hybrid progenies, the expression levels of a series of genes involved in VOC biosynthesis were detected (Figure 4). Compared with the fragrant parenteral *A. ‘Mystral’*, the expression of *AaDXS*, *AaDXR,* and *AaCMK* were increased in the plant 08-380-20, which might lead to the production of terpenoid compounds. Other genes involved in the MEP, phenylpropane biosynthesis, or shikimate pathways showed similar expression patterns in plant 08-380-20 and *A.* *‘Mystral’*. Compared with *A. ‘Mystral’*, *AaEPSPS* and *AaPAL2* demonstrated increased expression in plant 08-377-9, which was related to the high amount of benzenoid compounds detected. On the other hand, the decreased expression levels of genes involved in the MEP pathway, including *AaDXS*, *AaDXR*, *AaMDS*, *AaHDS,* and *AaTPS*, may have led to the reduced content of terpenoids in plant 08-377-9. In plants 08-377-3 and 08-382-48, the expression levels of most VOC biosynthesis-related genes were decreased, which was consistent with the lower amount of floral scent compounds. These results indicate that the different content of floral scent compounds in the progeny plants of the performed cross may be the result of different expression levels of VOC biosynthetic pathway-related genes.

## 3. Discussion

The floral scent is a significant ornamental characteristic that improves the commercial value of ornamental plants [18]. *Anthurium* is an important commodity flower worldwide, however the odors of *Anthurium* range from pleasant to unpleasant. Therefore, it is of great significance to explore the mechanism of *Anthurium* floral scent production and to cultivate *Anthurium* cultivars with a pleasant fragrance.

In most plants, floral scent is produced by a series of chemical compounds, including terpenoids, phenylpropanoids/benzenoids, fatty acid derivatives, carotenoid derivatives, and sulfur- or nitrogen-containing compounds. In this study, the scents of *A.* *‘Mystral’* were found to be composed of two major compounds: terpenoids (70%) and benzenoids (28.5%). Among these compounds, eucalyptol and acetic acid, phenylmethyl ester were the predominant VOCs, accounting for 57.5% and 23.2% of total VOCs, respectively. In the F1 hybrids of *A. ‘Mystral’* and *A.* ‘Alabama’, two plants (08-377-09 and 08-382-20) with strong fragrance and two plants (08-377-09 and 08-382-20) with weak fragrance were chosen to identify different VOC contents in these two groups of plants. In plant 08-377-09, acetic acid, phenylmethyl ester accounted for 79.6% of the total VOCs and the percentage of eucalyptol was 3.8%. In plant 08-382-20, eucalyptol was the major volatile compound and no acetic acid, phenylmethyl ester was detected. Interestingly, the total amounts of VOCs in plants 08-377-09 and 08-382-20 were very low, but acetic acid, phenylmethyl ester (80.5%) and eucalyptol (60.5%) were still the major volatile compounds. These results indicated the major volatile compounds of the F1 hybrid progeny were consistent with the fragrant parent.

A previous survey of floral scent in 147 *Anthurium* species and hybrids showed that most plants emitted scent only in the morning (45%) and at the pistillate stage (77%) of floral development [37]. For *A.* *‘Mystral’*, the floral scent lasted from morning to afternoon and spadix samples at the pistillate stage of development, between 10:00 a.m. and 12:00 p.m., were taken for GC-MS analysis. For qPCR analysis, the expression levels of floral scent biosynthesis-related genes were compared across the top, middle, and base parts of the spadix. The qPCR results showed that most genes exhibited their highest level of expression in the top of the spadix (Figure 3). Thus, the top part was chosen to test the expression level of floral scent biosynthesis-related genes in the F1 hybrids (Figure 4).

The inheritance of floral scent was complex, and was not simply controlled by nuclear inheritance or cytoplasmic inheritance. Our results demonstrated that having a fragrance-free parent can lead to progeny with strong fragrance, which indicates that cytoplasmic inheritance is not essential for the fragrance trait but may increase the frequency of scented progeny. In addition, several studies have demonstrated that the relationship between flower color and scent are partly linked by inheritance, as the two traits rely on shared biosynthetic pathways [38]. To explore the relationship between spathe color and spadix scents in *Anthurium andraeanum*, hybridization experiments were carried out using *A.* *‘Mystral’* (with red spathes, white spadix, and strong fragrance) and *A.* ‘Alabama’ (with white spathes, pink spadix, and no fragrance) as parents. Among 226 F1 hybrid individuals from the cross combination (*A. ‘Mystral’* (♀) × *A.* ‘Alabama’ (♂)), 170 of 220 plants with red spathes emitted floral scent at different times of the day, and 135 of the 170 plants had white spadix. For the remaining six plants with white spathes, three individuals emitted floral scent with white or pink spadix. A similar phenomenon was also observed in the F1 hybrids from the reciprocal cross combination (*A.* ‘Alabama’ (♀) × *A. ‘Mystral’* (♂)). The results suggested that there was no obvious relationship between the floral scent and flower color in *Anthurium andraeanum*.

In recent years, terpenoid and phenylpropanoid/benzenoid biosynthetic pathways have been well characterized, but less was known about the biosynthesis of fatty acid derivatives [23]. In *A.* *‘Mystral’* and the F1 hybrids, monoterpenes and benzenoids were the major floral scent compounds and the transcript levels of key enzyme genes that are involved in the biosynthetic pathways of these two compounds were identified. In the existing database of *Anthurium*, we found six key enzymes in the MEP pathway, including DXR, CMK, MDS, HDS, and TPS. Our results revealed that all six genes exhibited extremely higher expression in *A.* *‘Mystral’* than in *A.* ‘Alabama’. This extreme difference was consistent with the great difference in floral scent content. Compared with *A.* *‘Mystral’*, the content of terpenoids was relatively higher in plant 08-382-20, and three of six key enzyme genes (*AaDXS, AaDXR,* and *AaCMK*) were upregulated. These results indicated that *AaDXS*, *AaDXR*, and *AaCMK* were much more important in terpenoid synthesis, but these results might be influenced by other factors such as different development stages and different inflorescence parts. By comparing the content of benzenes and the expression of related genes in *A.* *‘Mystral’* and the progenies, *AaEPSPS* and *AaPAL2* were identified as more important enzymes in the biosynthesis of benzenoids. In conclusion, the expression patterns of genes related to floral scent synthesis were consistent with the relative contents of different types of floral scent components.

## 4. Materials and Methods

### 4.1. Plant Materials and Growth Conditions

*A. ‘Mystral’* (with strong fragrance) and *A.* ‘Alabama’ (with no fragrance), were obtained from Guangzhou Flower Research Center (Guangzhou, China) (Figure 4). Plant group 08-377 were the F1 generation of individual plants from the cross of *A.* *‘Mystral’* (♀) × *A.* ‘Alabama’ (♂), while 08-382 were the F1 generation of individual plants from the reciprocal cross of *A.* ‘Alabama’ (♀) × *A. ‘Mystral’* (♂). All plants were grown in the greenhouse at 23–28 °C and 80% relative humidity, under a natural photoperiod. To investigate the formation and regulation of floral scent during different reproductive phases, the development of spadices were divided into three stages: S1, spathe folding stage; S2, pistillate emerge stage; and S3, spadix fully extended stage. The spadices at S2 stage, which were collected between 10:00 a.m. and 12:00 p.m., were used for the analysis of volatile compounds.

### 4.2. Extraction and Determination of Volatile Compounds

In *Anthurium*, floral scents are mainly emitted at stage S2. Thus, the spadices at stage S2 were harvested for volatile constituent analysis, according to Yue et al. [39]. The spadix was enclosed in a 500 milliliter (mL) glass bottle with the addition of 1.728 micrograms (μg) ethyl caprate, which served as an internal standard. After waiting 30 min to achieve equilibrium, a polydimethylsiloxane fiber (PDMS, with 50/30 micrometer (μm) divinylbenzene/Carboxen) fiber (Supelco) was used to collect volatiles. Then, the collected volatiles were detected by GC-MS using an Agilent 7890A GC and Agilent 5975C MSD. The instrument was equipped with an Agilent HP-5MS capillary column (30 m × 0.25 mm) and helium was used as a carrier gas at a constant flow of 1.0 mL/min. The oven temperature was initially maintained at 45 °C for 2 min, followed by an increase of 5 °C/min until it was finally maintained at 250 °C for 5 min. Identification of individual compounds was performed by the comparison of mass spectra and retention times with authentic standards, or with the NIST 08 mass spectra library. Quantification was based on peak areas and the quantity of internal standard using the Agilent ChemStation Data Analysis Application. The relative content of aroma components was calculated as follows: the mass of compound (μg·gFW^−1^·h^−1^) = mass of internal standard × area under the peak of a compound/area under peak of internal standard/fresh weight of sample.

### 4.3. Analysis of Quantitative Real-Time PCR (qRT-PCR)

To evaluate the transcript profiles of floral scent-related genes, the spadices at stage S2 were harvested for RNA extraction. Total RNA was extracted from the above samples using TRIzol Reagent (Invitrogen, Carlsbad, CA, USA) following the manufacturer’s protocol. First-strand cDNAs were synthesized from 1.0 μg total RNA using the HiScript II RT-PCR system (Vazyme, Nanjing, China), according to the manufacturer’s instructions. Q-PCR reactions (20 microliter (μL) volume containing 1.0 μL cDNA as the template) were performed using the CFX connect Real-Time PCR System (Bio-Rad, Hercules, CA, USA) in standard mode with the KAPA SYBR FAST Universal qRT-PCR Kit (Kapa Biosystems, Wilmington, MA, USA). *Anthurium Actin* was used as the internal reference gene to quantify the relative expression level of target genes [40]. Primer sequences for qPCR were designed using NCBI Primer-BLAST, and are listed in Supplementary Table S1. The relative expression levels of target genes were calculated by the 2^−ΔΔCT^ method. Three biological replicates were performed per experiment.

### 4.4. The Olfactory Tests of the Hybrid Progenies

The olfactory tests of hybrid progenies were carried out in the greenhouse of the Guangzhou Flower Research Center. Inflorescences with two or three mature pistils were selected as the test materials. The experiment was performed by treated individuals between 10:00 a.m. and 12:00 p.m. in the morning and was repeated six times. “Strong fragrant” indicated the experimenters could easily smell a strong fragrance, and the results of six repetitions were consistent; “weak fragrant” indicated the experimenters could smell a faint fragrance and the results of four repetitions were consistent at least; “no fragrant” indicated the experimenters could not smell any fragrance, and the results of six repetitions were consistent. 

## 5. Conclusions

The floral scent profile of *A.* *’Mystral’* was found to be dominated by terpenes (70%), mostly eucalyptol. The scent profile also contained smaller quantities of phenylpropanoid/benzenoids (28.5%), mostly phenylmethyl ester. The main components of the progenies with aroma, which were similar to those of *A.* *‘Mystral’*, were eucalyptol and phenylmethyl ester. By integrating volatile profiles with gene expression analysis, we could infer that variations in the relative proportions of *DXS*, *EPSPS*, and *PAL* genes and volatile compounds may alter the floral scent profile in *Anthurium*. Further studies will aim to analyze the location, sequences, and upstream sequences of these VOC biosynthesis-related genes, which may provide better understanding of the genetic underpinnings of floral scent and inheritance in *Anthurium*.

## Figures and Tables

**Table 1:** Relative amounts of volatile compounds identified in *A.* *‘Mystral’* and *A.* ‘Alabama’.

No.	Compounds	Molecular Formula	RT ^1^ (min)	Content (μg·gFW·h^−1^) ^2^ ± SD ^3^	
				*A. ‘Mystral’*	*A.* ‘Alabama’
	**monoterpenes**				
1	Eucalyptol	C_10_H_18_O	10.211	49.2 ± 2.8	-
2	*α*, *α*-4-trimethyl-3-Cyclohexene-1-methanol	C_10_H_18_O	14.182	7.07 ± 0.6	-
3	*β*-Pinene	C_10_H_16_	8.62	1.313 ± 0.4	-
4	*β*-Phellandrene	C_10_H_16_	8.551	0.566 ± 0.0	-
5	1-methyl-4-(1-methylethylidene)-cyclohexene	C_10_H_16_	11.876	0.414 ± 0.1	-
6	(*E*)-1,3,6-Octatriene, 3,7-dimethyl-	C_10_H_16_	10.691	0.232 ± 0.1	-
7	*α*-Pinene	C_10_H_16_	7.436	0.212 ± 0.0	- ^4^
8	*β*-Myrcene	C_10_H_16_	9.026	0.202 ± 0.1	-
9	4-methyl-1-(1-methylethyl)-3-Cyclohexen-1-ol	C_12_H_20_O_2_	14.474	0.152 ± 0.2	-
10	*cis*-2-Cyclohexen-1-ol,2-methyl-5-(1-methylethenyl)-, acetate	C_12_H_18_O_2_	18.874	0.121 ± 0.0	-
11	Thujone	C_10_H_16_O	12.711	0.061 ± 0.0	-
12	3-methyl-6-(1-methylethylidene)-cyclohexene	C_10_H_16_	13.592	0.051 ± 0.0	-
13	2,6-Octadienoic acid, 3,7-dimethyl-, methyl ester	C_11_H_18_O_2_	18.49	0.03 ± 0.0	-
	**sesquiterpenes**				
14	1H-Cyclopropa[*a*]naphthalene,1a,2, 3,3a,4,5,6,7b-octahydro-1,1,3a,7-tetramethyl	C_15_H_24_	21.454	0.172 ± 0.0	-
15	*γ*-Himachalene	C_15_H_24_	22.576	0.121 ± 0.0	-
	**phenylpropanoid/benzenoids**				
16	Acetic acid, phenylmethyl ester	C_9_H_10_O_2_	14.19	19.835 ± 1.5	-
17	Benzoic acid, methyl ester	C_11_H_12_O_2_	12.059	3.848 ± 0.5	-
18	Benzaldehyde	C_7_H_6_O	8.242	0.212 ± 0.1	-
19	2-Propenoic acid, 3-phenyl-, methyl ester	C_16_H_14_O_2_	20.07	0.131 ± 0.0	-
20	Indole	C_8_H_7_N	17.672	0.131 ± 0.2	-
21	Butylated Hydroxytoluene	C_15_H_24_O	23.337	0.091 ± 0.0	-
22	1-ethyl-2,4,5-trimethyl-, Benzene	C_11_H_16_	18.227	0.081 ± 0.0	-
	**Others**				
23	Tetradecane	C_14_H_30_	20.453	0.242 ± 0.2	0.226 ± 0.2
24	Heptadecane,2,6,10,14-tetramethyl	C_21_H_44_	22.015	0.242 ± 0.2	0.183± 0.2
25	Pentadecane	C_15_H_32_	22.948	0.242 ± 0.2	0.279± 0.2
26	1,2-Benzenedicarboxylic acid, butyl 2-ethylhexyl ester	C_16_H_22_O_4_	32.011	0.232 ± 0.1	-
27	2,6,10-trimethyl-Dodecane	C_15_H_32_	19.852	0.1 ± 0.0	0.082 ± 0.0
28	Cyclohexasiloxane, dodecamethyl-	C_12_H_36_O_6_Si_6_	18.628	0.081 ± 0.0	0.059 ± 0.0
29	decamethyl-cyclopentasiloxane	C_10_H_30_O_5_Si_5_	13.844	0.051 ± 0.0	-
30	Tridecane	C_13_H_28_	17.832	0.051 ± 0.0	-
31	10-Methylnonadecane	C_20_H_42_	19.692	0.04 ± 0.0	-
32	2,6,11,15-tetramethyl-Hexadecane	C_20_H_42_	26.415	0.04 ± 0.0	0.054 ± 0.0
33	3,5-dimethyl-Undecane	C_13_H_28_	26.518	-	0.055 ± 0.0
34	Hexadecane	C_16_H_34_	27.777	-	0.103 ± 0.1
				85.567	1.041

^1^ RT, retention time; ^2^ the mass of compound (μg·gFW^−1^·h^−1^) = mass of internal standard × area under the peak of a compound/area under peak of internal standard/fresh weight of sample; ^3^ all data are presented as mean ± standard error (*n* = 3); ^4^ indicates not detected.

**Table 2:** Olfactory test of F1 hybrids from two-hybrid combinations.

Hybrid Combinations	No. of Plants (Strong Floral Scent)	No. of Plants *(Weak Floral Scent)*	No. of Plants (Fragrance Free)	Ratio (No. Strong/No. Fragrance Free)
*A. ‘Mystral’* ♀ × *A. *‘Alabama’ ♂ (08-377)	60	210	93	3:1
*A.* ‘Alabama’ ♀ × *A. ‘Mystral’* ♂ (08-382)	6	48	37	3:2

**Table 3:** Relative amounts of volatile compounds identified in F1 hybrids.

No.	Compounds	Molecular Formula	RT ^1^ (min)	Relative Amount (μg·gFW·h^−1^) ^2^ ± SD ^3^			
				08-377-9	08-377-3	08-382-20	08-382-48
				Strong	Weak	Strong	Weak
	**Monoterpenes**						
1	*α*-Pinene	C_10_H_16_	7.436	0.342 ± 0.0	- ^4^	1.424 ± 0.1	1.048 ± 0.1
2	*β*-Pinene	C_10_H_16_	8.62	0.467 ± 0.1	-	0.937 ± 0.1	-
3	*β* -Phellandrene	C_10_H_16_	8.694	-	-	2.13 ± 0.2	0.382 ± 0.1
4	4-methylene-1-(1-methylethyl)-cyclohexene	C_10_H_16_	8.78	-	-	-	1.214 ± 0.1
5	Eucalyptol	C_10_H_18_O	10.199	4.215 ± 0.3	-	37.497 ± 1.3	11.49 ± 0.6
6	3,7-dimethyl-1,6-Octadien-3-ol	C_10_H_16_O	12.225	-	-	27.09 ± 11.0	-
7	(*E*)-1,3,6-Octatriene, 3,7-dimethyl-	C_10_H_16_	10.691	0.023 ± 0.0	-	0.109 ± 0.1	-
8	*cis*-Linalol oxide	C_10_H_18_O_2_	11.441	-	-	0.803 ± 0.1	-
9	1-methyl-4-(1-methylethylidene)-cyclohexene	C_10_H_16_	11.882	0.023 ± 0.0	-	-	-
10	*cis*-Limonene oxide	C_10_H_15_O	13.198	0.023 ± 0.0	-	0.56 ± 0.0	0.077 ± 0.0
11	*trans*-Limonene oxide	C_10_H_15_O	13.329	2.154 ± 0.2	0.013 ± 0.0	11.124 ± 0.6	1.265 ± 0.1
12	4-methyl-1-(1-methylethyl)-3-Cyclohexen-1-ol	C_10_H_18_O	14.485	-	-	-	-
13	*α*,*α*-4-trimethyl-3-Cyclohexene-1-methanol	C_10_H_18_O	14.868	0.296 ± 0.0	-	5.72 ± 0.6	-
14	4-methyl-1-(1-methylethenyl)-Cyclohexene	C_10_H_16_	14.971	0.308 ± 0.0	-	1.4 ± 0.1	-
15	*trans*-2-methyl-5-(1-methylethenyl)-Cyclohexanone	C_10_H_16_O	15.057	2.37 ± 0.2	-	10.309 ± 0.9	0.226 ± 0.0
16	2-methyl-5-(1-methylethenyl)-2-Cyclohexen-1-ol	C_10_H_18_O	15.269	0.057 ± 0.0	-	0.596 ± 0.1	-
17	(*S*)-2-methyl-5-(1-methylethenyl)-2-Cyclohexen-1-one	C_10_H_18_O	16.367	0.103 ± 0.0	0.011 ± 0.0	1.789 ± 0.5	1.2980.1
18	*trans*-Carvone oxide	C_10_H_14_O	16.934	1.425 ± 0.1	-	10.126 ± 0.6	0.703 ± 0.0
19	*cis*-2-Cyclohexen-1-ol,2-methyl-5-(1-methylethenyl)-, acetate	C_12_H_18_O_2_	15.967	-	-	-	0.112 ± 0.0
	**Sesquiterpenes**						
20	(*S*)-1-methyl-4-(5-methyl-1-methylene-4-hexenyl)-Cyclohexene	C_15_H_24_	23.234	0.011 ± 0.0	-	-	-
	**Benzene**						
21	Benzyl Alcohol	C_7_H_8_O	10.308	-	0.035 ± 0.0	-	-
22	Benzoic acid, methyl ester	C_8_H_8_O_2_	12.082	-	0.148 ± 0.0	-	-
23	Acetic acid, phenylmethyl ester	C_9_H_10_O_2_	14.273	87.442 ± 1.9	6.956 ± 0.5	-	0.294 ± 0.0
24	Benzoic acid, ethyl ester	C_9_H_10_O_2_	14.285	-	0.014 ± 0.0	-	-
25	Propanoic acid, phenylmethyl ester	C_10_H_12_O_2_	16.728	0.011 ± 0.0	-	-	-
26	Butylated Hydroxytoluene	C_15_H_24_O	23.354	-	0.03 ± 0.0	-	-
27	Benzyl Benzoate	C_14_H_12_O_2_	29.133	4.809 ± 0.8	-	-	0.082 ± 0.0
28	Benzoic acid, 2-hydroxy-, phenylmethyl ester	C_14_H_12_O_3_	32.074	0.547 ± 0.0	-	-	-
	**Fatty Acid Derivatives**						
29	1-Butanol, 3-methyl-, acetate	C_7_H_14_O_2_	6.028	-	0.2066 ± 0.0	-	-
30	1,2-Benzenedicarboxylic acid, butyl 2-ethylhexyl ester	C_16_H_22_O_4_	32.097	-	-	0.316 ± 0.0	-
31	Phthalic acid, isobutyl octyl ester	C_20_H_30_O_4_	32.028	-	0.025 ± 0.0	-	0.233 ± 0.0
32	Hexanedioic acid, bis(2-ethylhexyl) ester	C_22_H_42_O_4_	34.958	4.627 ± 0.2	-	-	-
33	4-(2,6,6-trimethyl-1-cyclohexen-1-yl)-2-Butanone	C_13_H_22_O_2_	21.569	-	-	0.195 ± 0.0	-
	**Others**						
34	Tridecane	C_13_H_28_	17.832	0.034 ± 0.0	0.017 ± 0.0	-	-
35	Tetradecane	C_14_H_30_	20.459	0.125 ± 0.0	0.11 ± 0.0	0.207 ± 0.0	0.265 ± 0.0
36	Pentadecane	C_15_H_32_	22.942	0.148 ± 0.0	0.092 ± 0.0	0.256 ± 0.0	0.203 ± 0.1
37	Heptacosane	C_27_H_56_	22.238	-	-	-	-
38	Hexadecane	C_16_H_34_	25.3	0.034 ± 0.0	0.032 ± 0.0	-	-
39	2,6,10-trimethyl-Pentadecane	C_18_H_38_	27.669	-	0.02 ± 0.0	-	-
40	3-methyl-Tetradecane	C_15_H_32_	22.244	-	0.023 ± 0.0	-	-
41	5-methyl-3-Octyne	C_9_H_16_	17.346	-	-	0.207 ± 0.0	-
42	Cyclohexasiloxane, dodecamethyl-	C_12_H_36_O_6_Si_6_	18.628	0.034 ± 0.0	0.032 ± 0.0	0.11 ± 0.0	0.051 ± 0.0
43	10-Methylnonadecane	C_20_H_42_	19.515	0.023 ± 0.0	0.016 ± 0.0	-	-
44	3-methyl-Tridecane	C_14_H_30_	19.709	-	0.012 ± 0.0	-	-
45	2,6,10-trimethyl-Dodecane	C_15_H_32_	19.852	0.046 ± 0.0	0.14 ± 0.0	0.073 ± 0.0	0.056 ± 0.0
46	3-cyclohexyl-Decane	C_16_H_32_	21.683	-	0.03 ± 0.0	-	-
47	Heptadecane,2,6,10,14-tetramethyl	C_21_H_44_	22.021	0.114 ± 0.0	-	0.158 ± 0.0	-
				109.811	7.9626	113.137	18.999

^1^ RT, retention time; ^2^ the mass of compound (μg·gFW^−1^·h^−1^) = mass of internal standard × area under peak of a compound/area under peak of internal standard/fresh weight of sample; ^3^ all data are presented as mean ± standard error (*n* = 3); ^4^ indicates not detected.

## Data Availability

The data presented in this study are available on request from the corresponding author.

## Supplementary Materials

Table S1: Primer Sequences for qPCR.

## Sample Availability

Samples of the compounds are available from the authors.
